# Meta-Analysis on the Associations of TLR2 Gene Polymorphisms with Pulmonary Tuberculosis Susceptibility among Asian Populations

**DOI:** 10.1371/journal.pone.0075090

**Published:** 2013-10-04

**Authors:** Jia-Jia Wang, Xian Xia, Shai-Di Tang, Jie Wang, Xiao-Zhao Deng, Yun Zhang, Ming Yue

**Affiliations:** 1 Department of Epidemiology and Biostatistics, School of Public Health, Nanjing Medical University, Nanjing, Jiangsu, China; 2 Department of Nosocomial Infection Control, General Hospital of Beijing Military Region, Beijing, China; 3 Department of General Practice, Kangda College, Nanjing Medical University, Nanjing, Jiangsu, China; 4 Institute of Disease Control and Prevention, Huadong Research Institute for Medicine and Biotechnics, Nanjing, Jiangsu, China; 5 Institute of Epidemiology and Microbiology, Huadong Research Institute for Medicine and Biotechnics, Nanjing, Jiangsu, China; 6 School of Life Science and Technology, China Pharmaceutical University, Nanjing, Jiangsu, China; Centers for Disease Control and Prevention, United States of America

## Abstract

**Background:**

Publications regarding the associations of toll-like receptor 2 (TLR2) G2258A and T597C polymorphisms with pulmonary tuberculosis (PTB) susceptibility are inconsistent. A meta-analysis was conducted to investigate the relationship between TLR2 G2258A and T597C polymorphisms with PTB susceptibility.

**Methods:**

A systematic search was performed for published studies on the relationship between TLR2 polymorphisms and PTB susceptibility. Information was gathered from each eligible study, and statistically analyzed.

**Results:**

6 eligible studies, totaling 1301 cases and 1217 controls on G2258A genotypes, and 8 studies, totaling 2175 cases and 2069 controls on T597C genotypes, were included in the analysis. TLR2 2258G allele and 2258GG genotype were found to be associated with decreased PTB susceptibility (A vs. G: OR  = 3.02, 95% CI: 2.22–4.12, *P*<0.001, GA+AA vs. GG: OR  = 2.69, 95% CI = 1.49–4.87, P = 0.001). In the subgroup analyses, the 2258G allele and 2258GG genotype also exhibited a protective effect of PTB risk in Asians (A vs. G: OR  = 2.95, 95% CI: 1.91–4.55, P<0.001; GA+AA vs. GG: OR  = 3.59, 95% CI: 2.23–5.78, P<0.001), while no associations were observed in Caucasians. No significant associations between T597C polymorphism and PTB were found in the allele model (C vs. T: OR  = 0.95, 95% CI: 0.86–1.04, P = 0.28), co-dominant model (CC vs. TT: OR  = 0.88, 95% CI = 0.92–1.40, P = 0.25; CT vs. TT: OR  = 0.92, 95% CI = 0.80–1.06, P = 0.28), recessive model (CC vs. TT+TC: OR  = 0.96, 95% CI: 0.80–1.16, P = 0.69), or dominant model (TC+CC vs. TT: OR  = 0.93, 95% CI = 0.76–1.15, P = 0.51). The associations of T597C polymorphism with PTB susceptibility, in the ethnic-specific analyses, were still not significant.

**Conclusion:**

TLR2 2258G allele may provide protective effects against PTB susceptibility, particularly among Asians, whereas TLR2 T597C polymorphism might not be associated with PTB susceptibility.

## Introduction

Tuberculosis (TB), mainly caused by *Mycobacterium tuberculosis* (*M. tuberculosis*), leads to a considerable global health burden, especially in Southeast Asia [1]. More than one-third of the world's population has been infected with the *M. tuberculosis*, with 1% currently infected. However, only 10% of those who are infected advance to clinical diseases, such as pulmonary tuberculosis (PTB) [2]. The mechanism of host response to *M. tuberculosis* is still unclear. Until now, multiple factors have been reported to affect the outcome of *M. tuberculosis* infection, including age, gender, ethnicity, etc. The host genetic factors are assumed to play a critical role in tuberculosis pathogenesis, through impact on the gene expression of cytokines and chemokines, which are implicated in the host immune response. Furthermore, both animal models and segregation analyses have been performed to support the idea of genetic susceptibility to PTB [3]–[5]. A multitude of susceptibility gene research studies, as well as several genome-wide linkage scans [6]–[8], have identified the association of gene variants and PTB susceptibility, such as genes of TLRs, IL10, INF-γ, and HLA [9]–[12].

Toll-like receptors (TLRs), the vital pattern recognition receptors (PRRs), are composed of 13 trans-membrane proteins, and are mainly expressed in immune cells, such as dendritic cells and epithelial cells. TLRs play a vital role in the first line of host defense. They induce adaptive immune reactions against microbial pathogens [9], and thus are viewed as the key sensors of mycobacterial infections. TLR2, a key member of the TLRs family, could recognize a variety of bacterial lipoproteins, including the peptides derived from *M. tuberculosis*. After recognition of bacterial lipoproteins, TLR2 activates the MyD88 adaptor-like protein (Mal), and triggers a signaling pathway, which induces further immune response [13]–[14]. It has been supposed that single nucleotide polymorphisms (SNPs) of TLR2 gene are associated with PTB susceptibility. Several studies have explored the association between PTB susceptibility and SNPs of TLR2 gene, among which G2258A and T597C were the two most widely discussed SNPs. Lorenz et al. [15] found that the mutation of G2258A could decrease the response of macrophages to bacterial peptides, while Thuong et al. [16] reported that T597C might result in an attenuated early innate immune response to infection with *M. tuberculosis*.

Although previous studies have debated over the contribution of TLR2 G2258A and T597C polymorphisms to PTB, the small sample sizes and inadequate statistical power of these studies do not offer robust results. Therefore, we performed a meta-analysis to investigate the relationship between TLR2 G2258A and T597C polymorphisms with PTB susceptibility.

## Materials and Methods

### Search strategy

A systematic search was performed for published studies on the relationship between TLR2 polymorphisms and PTB susceptibility without language restriction. PubMed and Embase, as well as two Chinese databases (Wanfang and Chinese National Knowledge Infrastructure databases), were utilized to search the available articles published from January 2000 to March 2013. The search terms were as follows: “toll-like receptor 2” or “TLR2”, “polymorphisms” and “pulmonary tuberculosis”. The references of selected articles and review articles were also examined to identify additional eligible studies.

### Study selection

Inclusion criteria: (1) case-control studies, which evaluated the relationship between TLR2 G2258A and T597C polymorphisms with tuberculosis susceptibility; (2) available genotype frequencies from the studies; (3) original articles published in peer-reviewed journals. Exclusion criteria: (1) studies with non-specification of sample origins; (2) studies with insufficient or duplicate data; (3) studies with same author from similar origins.

### Data extraction

The following information was sought independently by two investigators from each eligible study according to the criteria listed above: first author's surname, year of publication, ethnicity, genotyping method, size of cases and controls, and size of cases and controls with TLR2 G2258A and T597C genotypes. For those studies that included subjects of PTB, extrapulmonary tuberculosis, and tuberculosis meningitis, only the numbers and genotype frequencies of PTB cases and healthy controls were extracted. For studies that included subjects of different ethnic groups, data were extracted for each group.

### Statistical analysis

The crude odds ratios (ORs) and 95% confidence intervals (95% CIs) were calculated to assess the association of TLR2 G2258A and T597C polymorphisms, with PTB risk for each study, based on extracted data. Subgroup analysis was also performed to assess the ethnic-specific effect. The chi-square based on Q statistic test was used for the assessment of heterogeneity and *P*<0.1 was considered statistically significant. When the effect was assumed to be lack of heterogeneity, the fixed-effects model (Mantel-Haenszel method) was used to calculate the pooled OR and 95% CIs; otherwise, the random-effects model (DerSimonian-Laird method) was used. Sensitivity analysis of the meta-analysis was performed by removing each single study step by step to assess the stability of the results. An estimate of potential publication bias was carried out by funnel plot when at least five papers were included. This was performed by plotting the log [OR] versus the standard error of log [OR] for each included study. Every circle dot represents a separate study for the indicated association. The perpendicular line represents the meta-analysis summary estimate. In the absence of publication bias, studies will be distributed symmetrically on the left and right of the perpendicular line. The funnel plot asymmetry was also assessed by Egger's regression and *P*<0.05 was considered representative of statistically significant publication bias. A chi-square test for goodness of fit was used to test Hardy-Weinberg equilibrium (HWE) in controls, and a *P*-value of <0.05 was considered significant. All analyses were done using Review Manager (v.4.2; Oxford, England). All *P*-values were two-sided.

## Results

### Study characteristics

**Figure 1:**
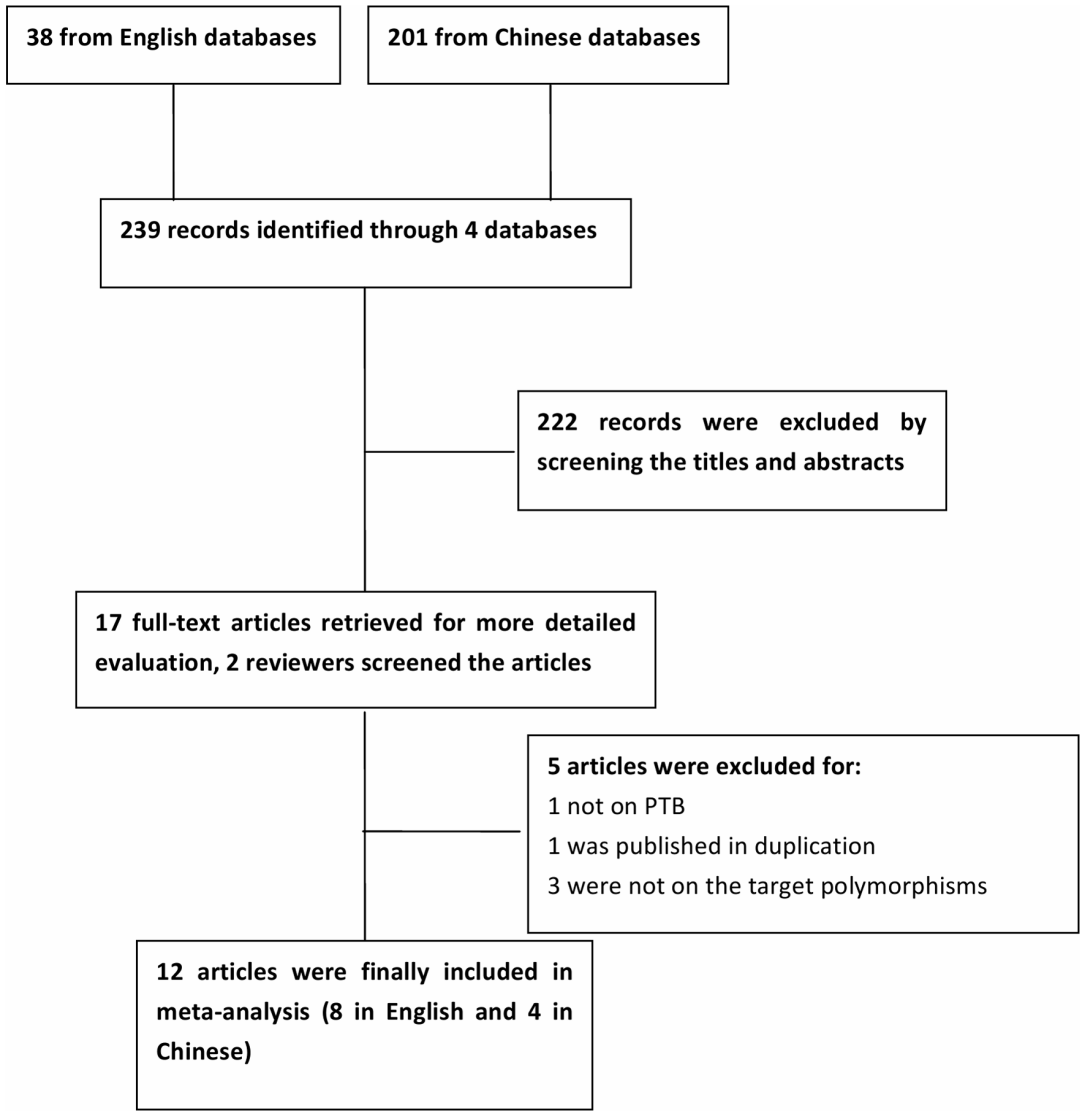
Flow diagram of selection process.

The search using PubMed, Embase, and two Chinese databases (Wanfang and Chinese National Knowledge Infrastructure databases) yielded 239 articles. Of these, 222 articles were excluded by reviewing the titles and abstracts. The remaining 17 articles were reviewed in detail and 5 of them were eliminated according to the inclusion and exclusion criteria. As a result, a total of 12 articles [9], [16]–[26] were identified. One article was considered as 3 separated studies, as it involved three different populations, and one study, conducted by Li in 2011, was a master dissertation. Overall, 6 eligible studies, totaling 1301 cases and 1217 controls on G2258A genotypes, and 8 studies, totaling 2175 cases and 2069 controls on T597C genotypes, were included in the analysis. A flow chart demonstrating the inclusion/exclusion of studies was displayed as Figure 1. The basic information, such as authors and published years, ethnicity, numbers of cases and controls, frequencies of various genotypes in PTB patients and healthy controls, genotyping methods and HWE in healthy controls of each study, were listed in Table 1 and Table 2. Genotype distributions in the control populations in two studies significantly deviated from HWE [9], [24].

**Table 1:** Characteristics of studies on the association of TLR2 G2258A polymorphisms with PTB.

Author, published year	Ethnicity	Genotyping methods	Case			Control			*P*_HWE_
			GG	GA	AA	GG	GA	AA	
Ogus 2004 [24]	Turkey (Caucasian)	PCR-RFLP	106	12	11	107	7	2	<0.001
Jin 2006 [23]	China (Asian)	PCR-SSP	99	71	0	168	31	0	0.233
Xue 2010 [20]	China (Asian)	Sequencing	204	1	0	202	1	0	0.971
Selvaraj 2010 [22]	Indian (Asian)	PCR-RFLP	192	1	0	198	1	0	0.972
Sanchez 2011 [17]	Colombia (Caucasian)	Mass-Array	463	3	0	296	4	0	0.907
Dalgic 2011 [21]	Turkey (Caucasian)	PCR-RFLP	100	38	0	186	14	0	0.608

PTB: pulmonary tuberculosis, HWE: Hardy-Weinberg Equilibrium.

**Table 2:** Characteristics of studies on the association of TLR2 T597C polymorphisms with PTB.

Author, published year	Ethnicity	Genotyping methods	Case			Control			*P*_HWE_
			TT	TC	CC	TT	TC	CC	
Thuong 2007 [16]	Vietnam (Asian)	Sequencing	95	73	11	205	154	18	0.105
Ma 2007 [9]	America (African American)	Sequencing	46	165	128	29	100	65	0.346
	America (Caucasian)	Sequencing	55	90	35	41	47	22	0.211
	America (Hispanics)	Sequencing	133	191	51	18	80	16	<0.001
Caws 2008 [25]	Vietnam (Asian)	Mass-Array	87	67	11	153	122	31	0.364
Xue 2010 [20]	China (Asian)	Sequencing	99	87	29	110	92	28	0.608
Che 2010 [19]	China (Asian)	Sequencing	52	54	9	68	68	20	0.644
Li 2011 [26]	China (Asian)	Sequencing	53	57	12	120	110	32	0.386
Sanchez 2011 [17]	Colombia (Caucasian)	Mass-Array	173	220	72	95	153	52	0.473
Shi 2012 [18]	China (Asian)	Sequencing	7	11	2	9	10	1	0.394

PTB: pulmonary tuberculosis, HWE: Hardy-Weinberg Equilibrium.

### Meta-analysis results

#### G2258A

Data could only be pooled in the allele and dominant models due to the rare frequency of the mutant homozygous genotype of G2258A in the included studies. In the allele model, the G allele was found associated with a lower PTB susceptibility (A vs. G: OR  = 3.02, 95% CI: 2.22–4.12, *P*<0.001) (Figure. S1). Similarly, the GG genotype significantly decreased PTB susceptibility in the dominant model (GA+AA vs. GG: OR  = 2.69, 95% CI = 1.49–4.87, *P* = 0.001) (Figure. S2).

Subgroup analyses were performed to assess the ethnic-specific PTB susceptibility in Asians and Caucasians. Significant associations were observed in Asians both in the allele model (A vs. G: OR  = 2.95, 95% CI: 1.91–4.55, *P*<0.001) and the dominant model (GA+AA vs. GG: OR  = 3.59, 95% CI: 2.23–5.78, *P*<0.001). However, no significant association was identified in Caucasians (G vs. A: OR  = 2.36, 95% CI: 0.93–6.04, *P* = 0.07; GA+AA vs. GG: OR  = 2.23, 95% CI: 0.75–6.59, *P = *0.15).

#### T597C

No significant associations were found between TLR2 T597C polymorphism and PTB susceptibility in the allele model (C vs. T: OR  = 0.95, 95% CI: 0.86–1.04, *P* = 0.28) (Figure. S3), the co-dominant model (CC vs. TT: OR  = 0.88, 95% CI = 0.92–1.40, *P* = 0.25; CT vs. TT: OR  = 0.92, 95% CI = 0.80–1.06, *P* = 0.28), the recessive model (CC vs. TT+TC: OR  = 0.96, 95% CI: 0.80–1.16, *P = *0.69), or the dominant model (TC+CC vs. TT: OR  = 0.93, 95% CI = 0.76–1.15, *P* = 0.51) (Figure. S4).

We further restricted the ethnic-specific analyses to Asians, and the associations of T597C polymorphism and PTB susceptibility were still not significant in the allele model (C vs. T: OR  = 0.99, 95% CI: 0.86–1.13, *P* = 0.84), the co-dominant model (CC vs. TT: OR  = 0.90, 95% CI = 0.66–1.24, *P* = 0.54; CT vs. TT: OR  = 1.05, 95% CI = 0.87–1.26, *P* = 0.62) the recessive model (CC vs. TT+TC: OR  = 0.88, 95% CI: 0.65–1.19, *P* = 0.41), or the dominant model (TC+CC vs. TT: OR  = 1.02, 95% CI: 0.86–1.22, *P* = 0.82).

### Sensitivity analyses and publication bias

**Figure 2:**
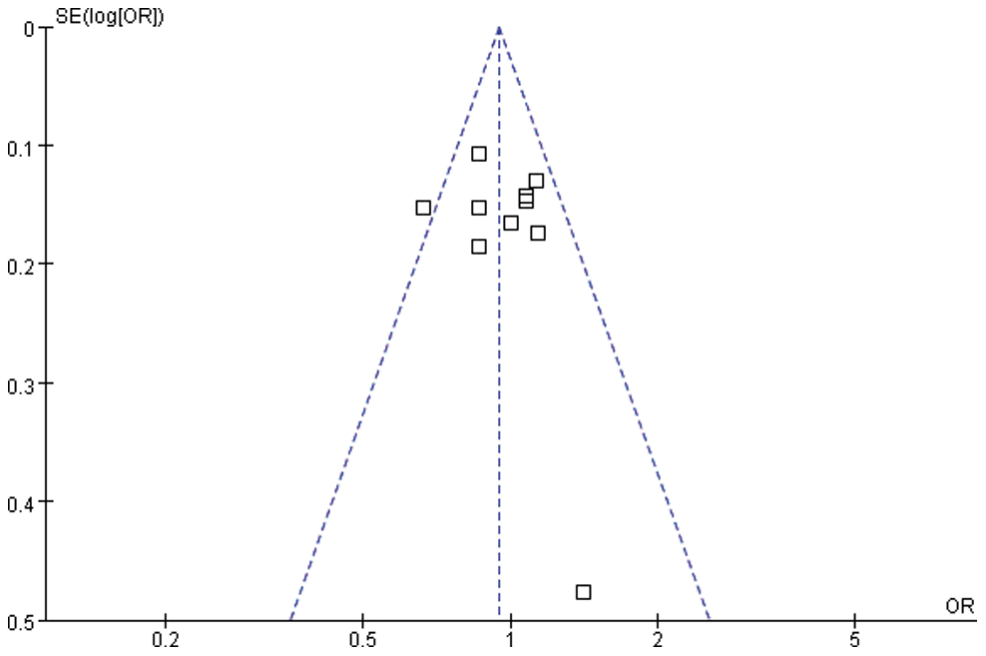
Funnel plot for the relationship between TLR2 T597C polymorphism and PTB susceptibility in the allele model.

Individual studies in the meta-analysis were sequentially deleted to reflect the influence of each study to the pooled OR. For all allele or genotype comparisons, the pooled ORs and 95% CIs were not qualitatively different (data not shown). The shapes of the funnel plots for these polymorphisms were symmetrical in all compared models (Figure. 2 shows funnel plot for T597C in the allele model). The results of the Egger's test did not suggest obvious publication bias for G2258A (*P* = 0.122 for A vs.G, *P* = 0.137 for GA+AA vs. GG). Similarly, no publication bias was detected for associations of T597C polymorphisms with PTB.

## Discussion

In this meta-analysis, we totally pooled 12 articles to evaluate the association of TLR2 G2258A and T597C polymorphisms with PTB susceptibility, and revealed that G2258A may contribute to PTB infection, while the T597C polymorphism showed no significant association with PTB susceptibility.

Both the innate and adaptive immune response determines the development and outcome of PTB. TLR2, a vital member of TLRs, has been considered to be involved in response to various bacterial lipoproteins, especially the 19-kDa lipoprotein from *M. tuberculosis* [27]. Moreover, studies, both *in vitro* and *in vivo*, revealed that TLR2 serves a critical role in the recognition of *M. tuberculosis* [28]. After stimulation of TLR2, the Mal was activated, followed by the stimulation of the transcription factor NF-κB [29], resulting in the regulation of the innate immunity. It has been reported that alveolar macrophages could sense the presence of Mycobacteria and kill the *M. tuberculosis* directly through the activation of TLR2 [30], [31]. Due to the wide effects of host genetic background on the immune response, the gene polymorphisms, which may alter the level of TLR2 and result in different outcomes of PTB, have gained increasing concern worldwide [32], [33]. Therefore, the TLR2 gene polymorphisms may influence the development and treatment response of PTB.

SNPs in the TLR2 gene might affect the transcription, synthesis, transport and secretion of TLR2, and consequently influence the occurrence, development, and outcomes of PTB. Accumulative evidence suggests that individuals carrying a defective TLR2 gene are more easily infected with *M. tuberculosis* [24], which implies that SNPs within the TLR2 gene might decrease the immune response, therefore, resulting in increased susceptibility to PTB. To support this hypothesis, several studies have been performed to investigate the relationship between TLR2 variants and PTB susceptibility in different populations [17]–[26], among which, G2258A and T597C have been most widely discussed. Compared to the wild-type, the 2258A was reported to have a significant decrease in NF-κB response against bacterial peptids in 293T cells transfected with wild-type or G2258A TLR2 constructs [15]. Due to the important function of G2258A polymorphism, multiple studies have been performed to understand the association of this SNP with PTB [17], [20]–[24]. Ogus et al. [24] first discussed the TLR2 G2258A variant in PTB and suggested that the G2258A polymorphism may influence the outcome of tuberculosis in Turks. However, no association between G2258A and tuberculosis was found in Indians [18]. The same research has also been carried out in different parts of China and had differing results [23]. Similarly, contradictive results have been found in other studies of T597C [17]–[21]. Thus, a meta-analysis could help strengthen the results of current studies and help draw more convincing conclusions.

It is important to note, we only pooled data in the allele and dominant models because of the rare frequency of mutant homozygous genotype of G2258A in the included studies. With an allele genetic model, this meta-analysis revealed a stronger association with PTB than with the dominant model (OR  = 3.02 vs. OR  = 2.69). This helped to conclude that G2258A carriage of mutant A allele significantly increased the PTB susceptibility, which is consistent with a previous study [15]. It is plausible that the A allele of G2258A may affect the transcription and expression of TLR2 and further affected the function of TLR2 protein. Further studies should focus on how the variant might impact gene expression and function. Besides, it has been demonstrated that population differentiation strongly influence the correlation between the TLRs polymorphisms and tuberculosis [34]. We thus restricted the race-specific analyses to performed a subgroup analysis, and found the G2258A polymorphism was strongly associated with PTB susceptibility in Asians, while no association was observed in Caucasians. These results might further confirm that population differentiation, such as genetic heterogeneity, plays a vital role in PTB susceptibility. For T597C, insignificant association of T597C polymorphism with PTB was identified in the allele model, co-dominant model, recessive model, and dominant model. One reasonable explanation might be that the heterozygous and mutant homozygous genotypes of T597C may have small effects on the influence of the immune function, which calls for further investigation.

There were also some limitations in our study. Firstly, the articles included in our meta-analysis were restricted to geographically distinct populations, thus limiting the generalizability of our conclusions. In addition, we were unable to obtain sufficient data related to age, gender, drinking, smoking and other factors, in order to perform a stratified analysis in this study. Secondly, the gene-gene and gene-environment interactions could not be taken into account in our analysis. Therefore, the relationship between TLRs polymorphisms and PTB should be confirmed in future studies.

## Conclusions

In conclusion, the result of our meta-analysis demonstrated that the TLR2 2258G allele may provide protective effects against PTB susceptibility, particularly among Asians. However, the TLR2 gene T597C polymorphism showed no significant association with PTB susceptibility. Although PTB is not solely a result of genetic factors, our conclusion would support the hypothesis that potentially functional polymorphisms in TLR2 may increase or decrease PTB and allow further investigation of TLRs variants for new therapies of PTB infection.

## Supporting Information

Figure S1 Forest plots of the association between TLR2 G2258A polymorphism and PTB susceptibility in the allele model.
(TIF)

Figure S2 Forest plots of the association between TLR2 G2258A polymorphism and PTB susceptibility in the dominant model.
(TIF)

Figure S3 Forest plots of the association between TLR2 T597C polymorphism and PTB susceptibility in the allele model.
(TIF)

Figure S4 Forest plots of the association between TLR2 T597C polymorphism and PTB susceptibility in the dominant model.
(TIF)

Checklist S1 PRISMA checklist.
(DOC)
