# Supplementary figures and images for: Meta-Analysis on the Associations of TLR2 Gene Polymorphisms with Pulmonary Tuberculosis Susceptibility among Asian Populations
Source: PLoS One. 2013 Oct 4;8(10):e75090. doi: 10.1371/journal.pone.0075090 (PMC3790778; doi:10.1371/journal.pone.0075090)

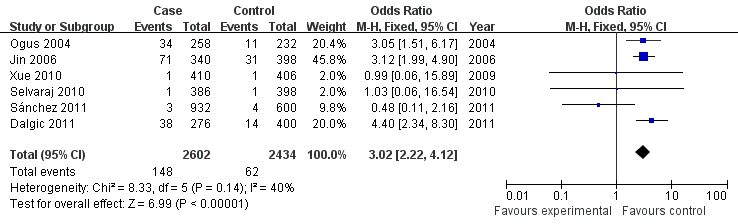

Supplement: Figure S1 — Forest plots of the association between TLR2 G2258A polymorphism and PTB susceptibility in the allele model. (TIF) [file pone.0075090.s001.tif]

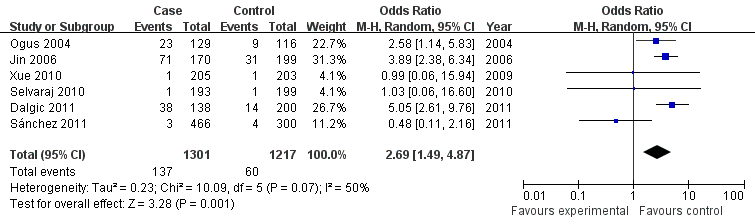

Supplement: Figure S2 — Forest plots of the association between TLR2 G2258A polymorphism and PTB susceptibility in the dominant model. (TIF) [file pone.0075090.s002.tif]

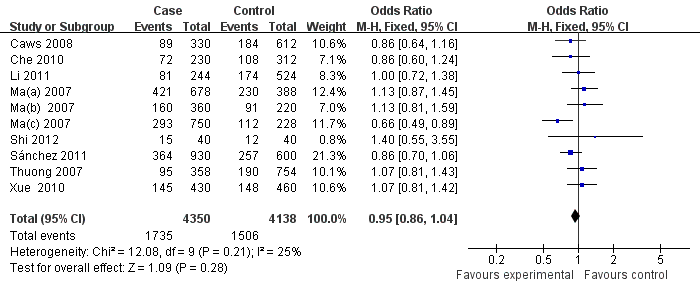

Supplement: Figure S3 — Forest plots of the association between TLR2 T597C polymorphism and PTB susceptibility in the allele model. (TIF) [file pone.0075090.s003.tif]

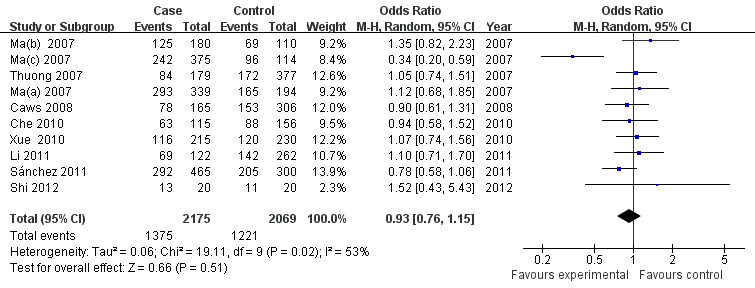

Supplement: Figure S4 — Forest plots of the association between TLR2 T597C polymorphism and PTB susceptibility in the dominant model. (TIF) [file pone.0075090.s004.tif]
